# Microfluidic-Based Nucleic Acid Amplification Systems in Microbiology

**DOI:** 10.3390/mi10060408

**Published:** 2019-06-19

**Authors:** Lena Gorgannezhad, Helen Stratton, Nam-Trung Nguyen

**Affiliations:** 1 Queensland Micro- and Nanotechnology Centre, Nathan Campus, Griffith University, 170 Kessels Road, Brisbane QLD 4111, Australia; lena.gorgannezhad@griffithuni.edu.au; 2 School of Environment and Science, Nathan Campus, Griffith University, 170 Kessels Road, Brisbane QLD 4111, Australia; h.stratton@griffith.edu.au

**Keywords:** bacterial nucleic acid, microfluidic, PCR, LAMP, droplet

## Abstract

Rapid, sensitive, and selective bacterial detection is a hot topic, because the progress in this research area has had a broad range of applications. Novel and innovative strategies for detection and identification of bacterial nucleic acids are important for practical applications. Microfluidics is an emerging technology that only requires small amounts of liquid samples. Microfluidic devices allow for rapid advances in microbiology, enabling access to methods of amplifying nucleic acid molecules and overcoming difficulties faced by conventional. In this review, we summarize the recent progress in microfluidics-based polymerase chain reaction devices for the detection of nucleic acid biomarkers. The paper also discusses the recent development of isothermal nucleic acid amplification and droplet-based microfluidics devices. We discuss recent microfluidic techniques for sample preparation prior to the amplification process.

## 1. Introduction

The steady increase of human life expectancy over the last century largely reflects the many advances in controlling bacterial infections. A range of areas in modern life, such as drinking and recreational water supply, industrial food production, pharmaceutical production, and point-of-care clinical diagnostics, require the identification of bacteria. Rapid and accurate bacterial detection is crucial for timely interventions including preventive vaccination and antimicrobial therapy [1]. However, conventional detection methods cannot entirely meet the need for rapid and accurate bacterial detection. For example, semi-quantitative plate culture has been the gold standard for bacterial identification, which is based on their morphological and metabolically characteristics. Although this method is well established and reliable, its major disadvantages are a long assay time, weak detection of non-culturable bacteria, low positive rate, and inefficient differentiation of bacteria at the level of strains or species [1,2].

More recently, state-of-the-art immunological and molecular diagnostics have been used for microbial detection. Immunological diagnostics are based on specific biomarker antibodies and optical sensing to detect the corresponding bacteria. Some drawbacks still exist with these methods, such as low or lack of signal, high background noise, and inconsistent results between replicate samples or controls [3,4]. Molecular diagnostics has been further expanded to proteomic and genomic methods such as mass spectrometry (MS), polymerase chain reaction (PCR), isothermal amplification, and high throughput next generation sequencing (NGS) [5,6,7,8].

MS has emerged as a powerful approach for analysing the molecular structure and molecular weight of analytes. Using the characteristic mass spectral fingerprints directly from intact bacteria, matrix-assisted laser desorption/ionization time-of-flight mass spectrometry (MALDI-TOF MS) has been used as a rapid method to identify the microbial species [9,10]. This technique can only identify highly abundant and purified bacterial samples, such as single colonies on a plate culture, and thus is still limited by the time-consuming procedure and low positive rate of bacterial culture [11].

PCR is one of the most well-known techniques in molecular biology for amplifying segments of DNA. Targeting the desired gene sequences, synthetic primers corresponding to these sequences can be designed to amplify the targets using the polymerase enzyme [12]. To implement conventional PCR, a number of repeated temperature cycles are needed. In contrast, isothermal amplification eliminates the thermo-cycling steps, reducing the cost and improving the overall assay quality. Therefore, isothermal amplification is more convenient than PCR for laboratories with limited technical capacity [13,14]. However, isothermal amplification has several challenges such as the dependence of sensitivity on annealing temperature, nonspecific amplification, RNase contamination, RNA with secondary structures or high guanine-cytosine (GC) content, PCR-borne mutations, pipetting errors, false negative results, cost, and time [15].

High-throughput NGS can be used to determine the whole genome of bacteria for highly veracious microbial typing [16] or determine the overall microbiota composition of a given sample without needing to culture the bacteria [17]. However, the huge amount of data produced by NGS leads to difficulties in managing and storing the results in clinical laboratories. Additionally, NGS approaches require enrichment, amplification, and labelling steps, causing the experiment to be both time- and cost-consuming, as well as increasing the possibility of false positive results [18]. Despite the broad use of traditional molecular techniques, there is still a research gap in the design, construction, and installation of de novo devices and pathways for improve and accelerate the detection of microbial nucleic acids.

In the past decade, microfluidic handling systems with microchambers and microchannels have been developed for a range of practical applications, particularly for nucleic acid (NA) analysis in microbiological assays [19,20]. Microfluidic devices have several benefits over conventional macroscale counterparts, such as laminar flow, the minute amount of reagents required, relatively fast mixing, low thermal inertia, and rapid heat transfer [21,22]. Due to the small size of microchannels, flow is mainly laminar and stable. Two or more streams flowing in a microchannel do not mix together. Without further engineering intervention, mixing relies entirely on molecular diffusion [23]. The small size and volume of microfluidic devices reduce the amount of sample and reagent in use, resulting in low operation cost [20]. The short diffusion length is another critical advantage of microfluidic tools as it improves reactions between enzymes and nucleic acids, thereby reducing assay time [24]. Heat and mass transfer can be well controlled in microfluidic devices. The small device size leads to a high surface to volume ratio and consequently a rapid mass and heat transfer rate. Thus, for a PCR process with many heating and cooling cycles, the total time can be significantly reduced [25].

In addition to the above features, distinct advantages, such as portability, automation, high throughput, and the ability to integrate multiple elements on a single chip, provide promising opportunities for on-chip analysis of microbial nucleic acids [26]. Various microfluidic chip-based portable nucleic acid analysers have been developed for the detection and identification of nucleic acids. Kopp et al. developed the first flow-through microfluidic PCR device based on silicon as the device material [27]. Glass and silicon were common materials for microfluidic devices in the early 1990s. Due to the relatively large design footprint and the corresponding cost, the device materials have been gradually shifting to polymers, such as polydimethylsiloxane (PDMS), polymethylmethacrylate (PMMA), and cyclic olefin copolymer (COC). The most recent PCR-based or isothermal amplification-based devices are composed of polymers. Whereas conventional PCR allows for the qualitative detection of particular NA sequences, microfluidic-based PCR allows for real-time and highly sensitive quantification of DNA down to the single molecule level, particularly with digital PCR using dispersed droplets as sample compartments. However, some bottlenecks still remain to be overcome [28].

Firstly, PCR-based microdevices require accurate thermal cycling of the sample. The fast temperature transition and the thermal homogeneity inside the PCR mixture critically affect the total run time, the number of thermal cycles, the efficiency, and the specificity of the amplification reactions. The requirement for accurate thermal control restricts the choice of materials for microfluidic devices, increasing their fabrication costs [29]. Secondly, during the amplification process, enzymes can be inhibited by a variety of compounds such as hemoglobin, humic acid, and cellular debris. Thus, microfluidic PCR systems require sample preparation steps, such as cell lysis and nucleic acid purification, to remove debris and unwanted compounds. Loop-mediated isothermal amplification (LAMP) avoids these issues and has been widely used for nucleic-acid-based amplification of a variety of pathogens. LAMP is highly sensitive, specific, and rapid, and is less susceptible to typical PCR inhibitors. The results generated by LAMP can be detected by the naked eye, and therefore do not need sophisticated instruments [30,31]. However, some bottlenecks still limit the application of LAMP-based microfluidics. Careful optimization of loop primers is necessary for reproducible and sensitive target detection. A relatively cold environment is needed for the experiments due to the instability of Bst Polymerase (a LAMP-specific enzyme) and deoxynucleotide triphosphate (dNTP) in warm and even room temperatures [32].

Recently, droplet-based microfluidics and digital microfluidics led to new developments of on-chip PCR devices. PCR within a single droplet is inexpensive, well-controlled, reproducible, and has high throughput as well as low contamination. Thus, droplet-based microfluidics has attracted considerable attention from the commercial world, particularly for digital PCR, which can be used to directly quantify and clonally amplify nucleic acids strands, making it more precise than PCR. In this approach, the sample is separated into a many partitions and the amplification is performed in each partition individually.

Digital microfluidics is a subclass of droplet-based microfluidics, dealing with discrete droplets. Digital microfluidics has huge potential due to its excellent capacity in running established chemistries and protocols in a single droplet. Splitting and merging of droplets in digital microfluidics allow for the implementation of sample preparation on the same platform [33,34]. There is interest in integrating various functions, such as sample preparation, amplification, and detection, in the same automated microfluidic device to reduce handling time and to prevent sample contamination [35,36]. Consequently, droplet-based microfluidics and particularly digital microfluidics play critical roles in designing advanced and robust nucleic acid amplification assays. Table 1 summarizes the existing relevant review papers, which mainly discuss microfluidic-based NA amplification in microbiology.

Although the majority of past review papers focused on just one microfluidics-based approach for NA amplification, no comprehensive review has covered all approaches, particularly for microbiology applications. The present paper provides a comprehensive overview of the most popular microscale NA amplification platforms for bacterial detection based on conventional PCR and LAMP. The paper introduces the seminal studies, followed by the latest advances in each field. Limitations and drawbacks of the techniques are discussed to identify research gaps with recommendations for future developments.

## 2. Spatial Polymerase Chain Reaction

If the sample moves continuously in a microchannel through regions with fixed temperatures, thermal cycling occurs spatially. Thus, this category is referred to as spatial or continuous-flow PCR devices. Advantages in heat transfer make this concept attractive for designing microfluidic PCR devices. Because only the sample mass receives periodic heating and cooling, the thermal inertia of the system decreases. The time interval between temperature transitions is therefore correlated with the sample flow rate and the thermal equilibrium time, enabling a quick thermal response and short reaction time [20,44]. Over the past decade, microfluidic devices for spatial PCR with serpentine and oscillating designs have been developed for rapid and simple detection of pathogenic bacteria.

### 2.1. Serpentine Design

**Figure 1:**
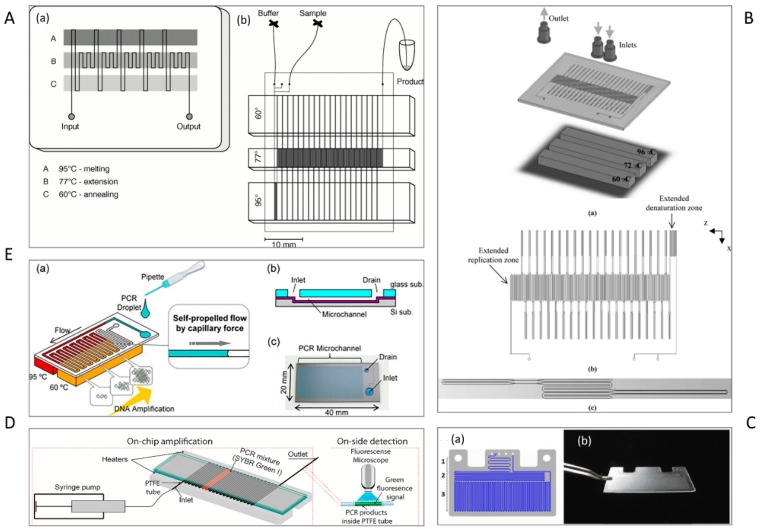
Dynamic PCR devices. (**A**) (a) Schematic illustration of a chip for flow-through PCR. Three temperature zones are stabilized at 95 °C, 77 °C, and 60 °C using thermostated copper blocks. The sample is pumped into a single channel etched in the glass chip. (b) Layout of the microfluidic device. The device has three inlets for carrying the sample/buffer and one outlet [27]. (**B**) (a) A schematic representation of the thermally-optimized 20-cycle continuous-flow PCR microfluidic device. (b) A top view of the microchip. (c) One cycle of the microchannel with different widths [47]. (**C**) (a) Schematic presentation of the chip. (1) Mixing zone. (2) Polymerase activation zone. (3) Thermal cycling zone. (b) Image of the chip [6]. (**D**) A schematic of on-chip amplification and on-site detection of amplicons using a GPG microdevice [52]. (**E**) Schematic illustration of a device for self-propelled continuous-flow PCR: (a) concept diagram, (b) cross-sectional view of device, and (c) picture of device [58]. Reproduced from the mentioned references with permission from the related journals.

Most spatial PCR devices use several heaters to provide multiple temperature regions for the corresponding PCR temperatures, i.e., initialization, denaturation, annealing, and extension regions [45]. In 1998, Kopp et al. reported the first attempt to amplify a 176-bp fragment from the DNA gyrase gene of *Neisseria gonorrhoeae* using a serpentine continuous-flow on-chip PCR [27]. Three copper blocks define three temperature zones that are maintained at 95 °C, 77 °C, and 60 °C (Figure 1A). The sample is pumped through a single serpentine channel etched in a glass chip, resulting in heating or cooling of the sample through the predefined temperature zones.

The channel geometry and the arrangement of the channel over the temperature zones are two critical parameters for optimising the total reaction time on the chip. At the fixed flow rate, the residence time of a given temperature zones is determined by the channel cross-section and the length of the channel section. For instance, with a fixed channel cross-section, the length of the extension region is designed to be longer than that of the denaturation or annealing regions [46] to increase the duration of the extension step in PCR. The transition time between two temperature levels is determined by the transition regions of the channel. To minimize the transition time, a smaller channel cross-section with a smaller width could be designed. Li et al. fabricated a PCR microdevice comprising a serpentine microchannel with various widths and a constant depth to amplify 90-bp *Bacillus anthracis* DNA fragments (Figure 1B). By changing the widths of the channel, the transitional time was decreased remarkably [47].

The other significant challenge of using serpentine channels for spatial PCR is heat management without thermal cross-talk. The devices need enough space between the heaters to provide sufficient thermal insulation, making the overall footprint relatively large. The longer channel over the narrow temperature zone requires extra loops, which also enlarges the footprint of the PCR device [46].

Since controlling multiple temperature zones on a single microfluidic device is challenging, reducing the required temperature zones and the number of heaters was a possible solution. Toward this idea, molecular-level interactions in various temperature zones have been investigated. Once the sample reaches the required temperature, the denaturation and annealing reactions occur almost immediately within one second, and the extension rate is on the order of 60–100 bases per second [48]. The investigation revealed that extension reactions even occur during the transition between annealing and denaturation temperatures. Thus, a holding time is not necessary if there are only a few amplification targets. Several studies on continuous-flow PCR with only two temperature zones have demonstrated rapid amplification cycle, high efficiency, high specificity, and low assay cost [49,50,51].

Fernández-Carballo et al. [6] reported a serpentine continuous-flow PCR with only two heaters below the chip (Figure 1C). Each heater consists of an aluminum heating block, a cartridge heater, a thermocouple, and a programmable temperature controller. The temperature control system was accompanied by an optical system for the real-time fluorescence detection of *Chlamydia trachomatis* and *Escherichia coli* O157:H7. The chip was designed with two inlets for the sample and the qPCR master mix, which are mixed in a long microchannel. The gradual movement of mixed reagents through serpentine channel leads to the reaction. First, the sample passed through a 95 °C zone for activation of polymerase and melting the DNA double strands. Next, the reagents were thermally cycled for 40 times between 95 °C and 62 °C for annealing, DNA synthesis, and denaturation. Recently, Trinh et al. [52] reported a serpentine continuous-flow PCR device composed of a glass-polytetrafluoroethylene (PTFE)-glass (GPG) sandwich and two heaters for denaturation and annealing/extension (Figure 1D). The GPG consists of two parallel glass slides holding a serpentine PTFE tube in-between. The chip is mounted on top of two heating copper locks. The glass slides are able to conduct the heat from the heaters to the PTFE microchannel without much loss. The GPG chip successfully amplified the specific sequences of *Salmonella* spp/ and *E. coli* O157:H7. Although the above-mentioned designs provided fast amplification reaction, simple fabrication, and portability for molecular detection of pathogens, further reduction to a single heater and creating the appropriate reaction regions for PCR are future remaining challenges [53].

For instance, Chen et al. [45] proposed the fabrication of a PDMS-based PCR device employing a single heater to detect *Coxiella burnetii*. By integrating one heater onto the chip centre and two heat pipes on the two sides of the chip, five temperature zones were created within the small footprint of the chip. The major achievement of this design is the significant reduction of the chip size and the thermal control cost. However, external and large pumps, such as syringe pumps, were necessary to continuously deliver the sample in all of the above-mentioned designs, leading to complex operations and preventing the further miniaturisation of the whole system. To solve this bottle neck, miniature pumping systems were integrated with the microfluidic devices [54,55]. Even though these micropumps efficiently controlled the sample flow, they still need external energy for running, which still causes a large overall footprint of the system. Passive capillary pumping could solve the problem of bulky power supply [56,57]. The capillary force of the liquid/air interface could be applied as a driving force for delivering the sample through microfluidic device without the need for an active micropump.

Tachibana et al. [58] designed a self-driven serpentine continuous-flow PCR device in a Si/glass microfluidic device for the amplification of 16S ribosomal DNA (rDNA) of *E. coli* (Figure 1E). The PCR solution only needs to be dropped onto the inlet, and then is autonomously transported by capillary force. The limitation of capillary-driven microfluidics is that both the capillary pressure at the fluid front and the viscosity of the solution change periodically due to the different temperature zones. This problem was solved by using an extremely long PCR microchannel. The length and design of the microchannel were optimised for the capillary pressure and the viscosity. In their further study, the team used a non-ionic surfactant for coating the wall of the microchannel to increase the capillary force, resulting in fast quantitative detection of *E. coli* and pathogenic *E. coli* O157 [59]. Most reported devices are similar to the original serpentine design of Kopp et al. [27].

### 2.2. Oscillating-Flow Design

Oscillatory microfluidics was introduced as the simplification of digital microfluidics. The oscillating-flow PCR method combines the cycling flexibility of stationary-chamber-based PCR and the fast dynamics of continuous-flow PCR [60]. Oscillating or bidirectional flow PCR was first described by Chen et al. in 2007 [61]. Since then, oscillatory-flow PCR has been one of the most favourable concepts due to advantages including simple system configuration, number/dwell time flexibility, simple application in real-time detection, large footprint reduction, and ability to amplify multiple samples in parallel [60].

In an oscillating-flow device, the PCR reagents are transported back and forth through a single channel spanning the various temperature zones. Due to the decreased surface inhibitory effect in oscillating-flow PCR, the channel is significantly reduced to a single straight channel, which only requires a smaller temperature zone and therefore keeps the device footprint to a minimum [62]. In the last decade, the oscillatory-flow PCR concept has been implemented in various substrate materials (silicon, glass, and polymers), with different heating (metal/ceramic heating blocks, Peltier heater, and film heaters) and pumping concepts (e.g., external pumps, and integrated micropumps) [60].

Oscillating-flow PCR devices still have shortcomings that limit their usage. For example, the concept only suits one DNA target in a single reaction solution; therefore, the detection throughput is low. Consequently, the detection speed is slow for multiple DNA targets. The concept is particularly not suitable for time-sensitive or throughput-sensitive diagnostics such as foodborne pathogens and related biowarfare agents.

**Figure 2:**
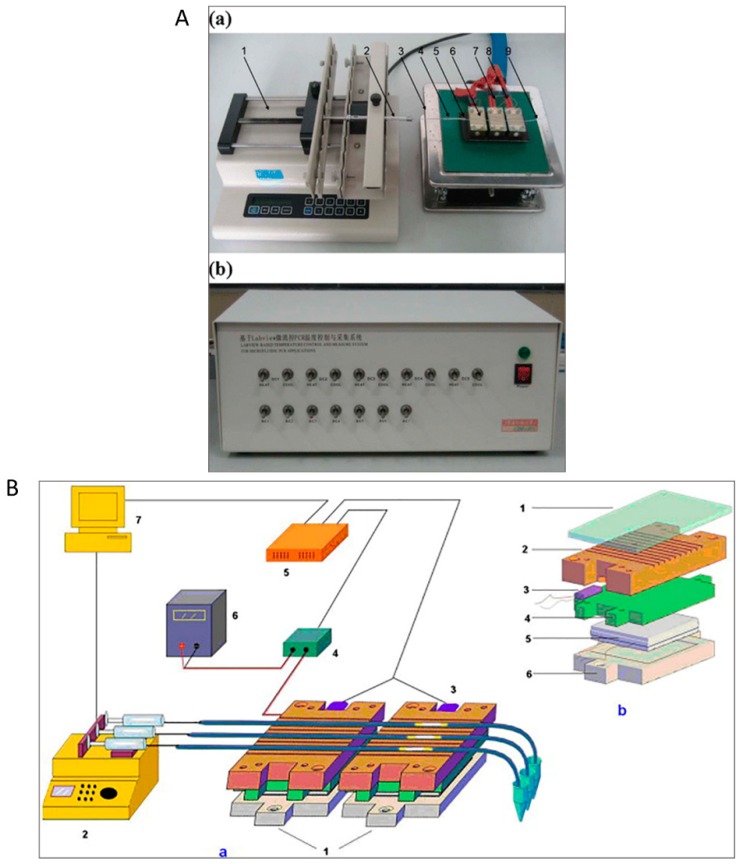
(**A**) Images of the oscillating-flow PCR microfluidic device. (a) The system consists of a precision syringe pump (1), glass syringe (2), lift table (3), silicon tube-based connector (4), support plate (5), copper block with a glass cover (6), thermocouple sensor (7), cartridge heater (8), and PTFE capillary tube (9). (b) The temperature control and measurement system [60]. (**B**) Schematic illustration of the oscillation-flow instrument. (a) Heating module (1), syringe pump (2), PT 100 sensor (3), relay (4), distributed multichannel controller (5), electric power source (6), and computer (7), and a magnified illustration of a heating module. (b) PC cover (1), grooved aluminum plate (2), PT 100 sensor (3), 6 aluminum plate holders (4), and Peltier heating element (5) [62]. Reproduced from the mentioned reference with permission from the related journal.

To overcome the throughput drawback, Zhang et al. [60] implemented oscillatory-flow multiplex PCR amplification in parallel in multiple channels, enhancing the detection throughput (Figure 2A). An array of independent parallel channels placed across the three temperature zones was able to perform multiplexed PCR of foodborne bacterial pathogens like *Listeria monocytogenes*, *E. coli O157:H7*, and *Salmonella enterica*. In another study, the oscillation-flow concept was implemented with a droplet reactor to achieve high-speed amplification (Figure 2B). Employing interfacial chemistry, a water-in-oil droplet was created by allowing an oil-water plug to flow through a polytetrafluoroethylene (PTFE) capillary. The resulting droplet serves as the reactor for oscillating-flow PCR and provides a stable reaction environment including fast reagent mixing and minimum surface adsorption. The efficiency of the device was evaluated using the amplification of the *New Delhi metallo-beta-lactamase* (*NDM-1*) gene [62].

## 3. Transient PCR

The transient approach introduces the PCR solution into a single or multiple reaction chambers. The chambers are then subjected to repeated heating and cooling processes corresponding to the thermocycling profile. Since the sample is stationary and its temperature varies over time, the amplification approach is referred to as transient PCR. In this class of PCR devices, the temperature profile does not depend on the channel design; thus, the heating and cooling protocols and consequently the PCR thermal cycling condition can be modified. Sample transport is not needed and therefore there is no necessity for pumps. However, this device cannot be operated continuously, so the sample throughput is restricted. The common transient PCR platforms for microbial NA analysis are centrifugal microfluidic devices, laboratory discs, and arrays.

### 3.1. Centrifugal Microfluidic Devices

Centrifugal microfluidics exploits centrifugal force and capillary force to control the liquid flow. Both forces are accessible as centrifugation is typically present in a rotating system and capillary force is dominant in microscale devices [63,64]. This group of devices are designed in the format of compact discs (CD) that house the reaction chambers and other components for the PCR [65]. The reaction mixture moves outward toward the edge of the disc, pushing air our, facilitating on-disc liquid storage [66]. Assembling CD-based microfluidics with on-board power provides the required thermal cycling for PCR [67].

Centrifugal microfluidics has several advantages over the conventional stationary microfluidics. First, the centrifugal driving mechanism provides a closed fluidic platform without the need for external pumps. Second, the elimination of any bubbles that may cause serious problems is particularly easy due to the scalable buoyancy in centrifugal microfluidics. Third, residual liquids that may be trapped due to surface forces can be easily removed from channels and chambers by adjusting the rotation speed. Forth, varying the rotation speed of the disc offers a combination of microfluidic sample preparation steps such liquid mixing, metering, aliquoting, switching, valving, and storage. These advantages of centrifugal microfluidic devices make them a potential candidate for bacteria detection [68].

**Figure 3:**
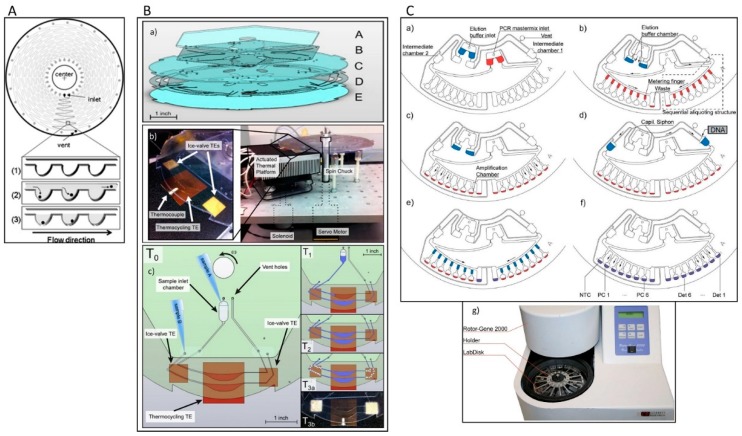
(**A**) Microfluidic design of a compact disc (CD) device and schematic illustration of single cell isolation: (1) a large number of microchambers align along a channel, (2) the cells flow through the microchambers and (3) are spread into individual microchambers [69]. (**B**) (a) Schematic graph of the multi-layered centrifugal disc. The disc is comprised of five layers of hard plastic: A. polycarbonate sheet, B. pressure sensitive adhesive tape (PSA), C. polycarbonate sheet, D. polycarbonate film, and E. polycarbonate film. (b) (Right) Image of the integrated centrifugal microfluidic platform for pumping, valving, and thermocycling of fluid. (Left) Close-up of the actuated thermal platform showing the location of the central thermocycling TE and two ice-valve TEs. (c) Schematic presentation of the hardware details and fluidic process for the integrated CD system [70]. (**C**) Schematic illustration of one microfluidic structure: (a) elution buffer and PCR mastermix are loaded to the inlets, (b) the elution buffer is transported into two elution buffer chambers while the PCR mastermix is distributed into metering fingers, (c) the PCR mastermix aliquots are gated into amplification chambers which filled with primers, (d) DNA is added to one of the sub-volumes, (e) each subvolume is aliquoted into several aliquots, (f) PCR is started, and (g) the Lab Disk is mounted to a custom-made holder [71]. Reproduced from the mentioned references with permission from the related journals.

Early centrifugal microfluidic CD-like devices were composed of silicon/glass with 24 serpentine channels and 313 microchambers (volumes of 1.5 nL) and were used for selective detection of *S. enterica* from a mixture of *S. enterica* and *E. coli*. The sample was placed in an inlet port and distributed over the microchambers by centrifugal force (Figure 3A). PCR was performed by thermocycling the entire disc. After PCR, the fluorescence intensity was evaluated by placing the device into an image analyser [69]. The inlet and outlet were sealed tightly with a butyl gum polymer, which prevents evaporation of the sample and effectively keeps the microchannel moist during the PCR cycles.

Amasia et al. reduced the evaporation in centrifugal microfluidic devices by freezing the liquid next to the PCR chamber and integrated pumping, valving, and thermoelectric heating/cooling in the system for the amplification and detection of *Bacillus anthracis* genes (Figure 3B). Three thermoelectric modules were used for thermocycling (one module) and for freezing small volumes of the PCR buffer in the channel network (two modules) to serve as ice valves. These ice valves were able to block the linkage channel between the PCR chamber and the outlet hole [70]. Although this method was successful at sealing the thermocycling chamber and preventing sample evaporation, controlling the temperature distribution between the thermoelectric heater and the ice-valve is challenging and requires complex design. In another attempt, Strohmeier et al. designed a cartridge for the detection of food borne pathogens such as *L. monocytogenes*, *Salmonella typhimurium*, enterohemorrhagic *E. coli*, *Staphylococcus aureus*, *Citrobacter freundii*, and *Campylobacter jejuni* [71]. This cartridge included qPCR reaction chambers for integrated positive and negative controls and was sealed with a pressure sensitive adhesive tape (Figure 3C). Czilwik et al. developed a passive microfluidic vapor-diffusion barrier to reduce pressure enhancement over PCR thermocycling [72].

The other critical component of this group of devices is the heater that regulates the temperatures for amplification. Heaters can be categorised as direct contact and non-contact types. Although many studies used direct contact heating to maintain the temperature for the amplification protocol [69,70,73], the elaborate designs and the rotating platform complicate their implementation. In contrast, non-contact heating allows the device to move while maintaining the temperature. Non-contact methods are based on microwave [74], magnetic induction [75], and infrared (IR) heating [76]. Microwave and induction heating need complex electronic components such as high frequency oscillators with high power inputs. IR heating has been used for rapid amplification of NA in microfluidic devices due to the quick heating and cooling of the sample [77]. Recently, a centrifugal microfluidic system with IR heating was developed for NA-sequence-based amplification reaction, targeting the tmRNA transcript of *Haemophilus influenzae*. In addition to the temperature control, this platform was also equipped with a control system for the rotation speed and positioning system of the reaction chamber for heating and fluorescence detection [66]. Table 2 provides an overview of centrifugal microfluidic platforms for the detection of various bacteria over the last few years.

### 3.2. Lab Disk

The further development of centrifugal microfluidics, particularly for bacteria detection, is integrating and automating the different analysis steps into a single device. This group of devices is known as “Lab Disk” [82]. A Lab Disk includes cell lysis, DNA extraction, amplification, and detection, in an integrated and automated format, to provide “sample in, answer out” for the identification of bacteria. Some pioneering platforms have already been developed for attaining this goal. For instance, Czilwik et al. [83] reported the integration of lysis, DNA extraction, and nested PCR in a single centrifugal microfluidic disk. Different types of bacteria (*Staphylococcus warneri*, *Streptococcus agalactiae*, *E. coli*, and *Haemophilus influenza*) were first lysed by adding chemical reagents. Next, binding, washing, and elution of DNA were conducted using magnetic beads. Lastly, the purified DNA was amplified and identified via nested real-time PCR. Despite simplifying the detection assays, the turnaround time of the complete analysis time was approximately 3 h and 45 min. Roy et al. [84] reported another device for the identification of *Bacillus atropheus* with integrated mechanical cell lysis, PCR, amplicon digestion, and microarray hybridization steps. The assay needed 2 h and 10 min to complete. With a single PCR chamber, this device was not suitable for multiplexing. Keller et al. reported multiplexed real-time PCR of *E. coli* employing a centrifuge-thermopneumatic fluid control system that uses the temperature-induced partial vapor pressure change of an enclosed gas volume for valving and aliquoting [82].

Despite the considerable progress in this field, improvement is still required. Yan et al. [85] recently developed a robust and user-friendly Lab Disk with a multiplexed detection ability of six types of bacteria. Compared to existing devices, this device has provided remarkable progress. For example, bacteria lysis is performed by only rotating a pair of magnets to generate bead-beating, while the chip remains stationary. The on-chip assay is rapid and the results can be interpreted through fluorescence detection or by the naked eye. Zhang et al. [86] developed a fully hand-powered centrifugal microfluidic platform for diagnostics of six pathogenic bacteria. This Lab Disk relies on zeolite-based purification of nucleic acids, loop-mediated isothermal amplification, and visual detection of the fluorescent signals. In addition, the flow actuation inside the device is enabled by a simple manual pull-out operation of the rack of the centrifuge, resulting in high-speed rotation of the disc and efficient mixing of preloaded sample/reagent fluids.

## 4. Array

Over the last decade, microarrays have been reported as a high-throughput platform for simultaneous detection of multiple gene targets [87]. The sensitivity of microarray technology is low, and compared to real-time PCR, the results are more variable [88]. Besides the microarray technology for parallel analyses of multiple bacteria, several designs have been created to integrate PCR with microarray techniques [89,90]. Most of these designs integrate solid-phase PCR with the microarray platform. However, solid-phase PCR is less efficient than solution-phase PCR [91]. Yauk et al. assessed genomic DNA microarray, amplified DNA microarray (PCR followed by microarray), and real-time PCR assays for their suitability for the identification of waterborne pathogens [88]. The attained results revealed that the real-time PCR assay is approximately 108 times more sensitive than the genomic DNA microarray and about 70 times more sensitive than the amplified DNA microarray [87,88]. Even though real-time PCR is still the gold standard for validation of the data generated by microarrays, it has limited capability to perform multiplex analyses of multiple samples and assays (<384 wells plates). To solve this problem, Roche Applied Science (Indianapolis, IN, USA) has commercialised the LightCycler^®^ 1536 Real-Time PCR System, uses utilises a 1536 multi-well plate. This system still needs a robotic liquid handling instrument to load the samples. Many efforts have been made to design PCR-based devices as a high-throughput platform for a larger number of samples.

Most of the first-generation devices need manual PCR mixture loading into individual reaction wells in a PCR array chip. For example, Nagai et al. developed a microchamber array in silicon for picoliter PCR using manual sample loading [92]. Solutions for avoiding manual sample loading include the expensive robotic liquid dispensing robot system or the immobilization of primers in a matrix [92,93,94]. Matsubara et al. used a nanoliter dispensing robotic system for loading PCR mixtures into a microchamber array [93]. In another work, primers were immobilized in a rectilinear array of 3072 holes in a stainless-steel plate. The array was then sealed inside a cassette using UV-curable epoxy [94]. This chip is commercially available from BioTrove (Open Array^®^ DLP Real-Time qPCR System, Woburn, MA, USA). This system could detect multiple bacteria from environmental water [95]. Although this platform offers a solution to high-throughput real-time PCR, loading samples to the device requires the differential surface treatment of the steel plate, making the chip fabrication complex and expensive.

Besides sample loading, the other significant challenge in designing these devices is sealing the chip. Uncured PDMS, mineral oils, pressure-sensitive adhesive tapes, and microvalves have been widely used for sealing microreactors [96,97,98]. Some PCR array chips have open or unsealed reactors to reduce the complexity of fabrication and operation [99,100]. Ramalingam et al. used open reactors for the detection of waterborne pathogens such as *Pseudomonas aeruginosa*, *Aeromonas hydrophila*, *Klebsiella pneumoniae* and *Staphylococcus aureus*. Recently, Fluidigm introduced a microarray for multiplex detection of bacteria, supporting a large number of simultaneous reactions with simple use [101,102].

## 5. Isothermal Amplification-Based Microfluidic Devices

Although PCR is the most popular approach for DNA amplification, the technique requires several thermocycles. Isothermal amplification of NA omits the thermocycling steps, resulting in low cost and high assay quality [38]. Over the last decade, a variety of isothermal methods have been developed for NA amplification such as loop-mediated isothermal amplification (LAMP), recombinase polymerase amplification (RPA), helicase-dependent amplification (HDA) [103], rolling circle amplification (RCA) [104], strand displacement amplification (SDA) [105], signal-mediated amplification of RNA technology (SMART) [106], nucleic acid sequence-based amplification (NASBA) [107], single primer-triggered isothermal amplification [108], and cross priming amplification (CPA) [109]. LAMP, RPA, and HDA have attracted significant attention from the microfluidics community.

### 5.1. RPA-Based Microfluidic Devices

Recombinase polymerase amplification (RPA) is a low temperature isothermal platform used to amplify target DNA consuming recombinase, a DNA polymerase, and DNA-binding proteins. The approach was first reported in 2006 by Piepenburg et al. [110]. Recombinase–primer complexes mediate primer binding at the specific sequence of double-stranded DNA and displacement of the non-template strand at low temperature (37 °C). The displaced new strand is maintained by single-strand DNA binding proteins and the primer is extended by DNA polymerase. The resulting double-strand DNA products can each be copied, leading to exponential amplification [38,111].

RPA provides significant advantages over existing amplification methods such as low incubation temperature, ease of primer design, sensitivity, and high tolerance to impurities in sample [112]. Due to the robustness of its biochemistry, RPA has been integrated into microfluidic devices. Lutz et al. developed a fully automated centrifugal microfluidic cartridge including pre-stored liquid and dry reagents for RPA [113]. The fluidic cartridge can run up to 30 reactions simultaneously in separate 10-mL microchambers. This system was employed for the detection of the antibiotic resistance gene *mecA* of *S. areus*. The limit of detection (LOD) was less than 10 copies and the assay time was approximately less than 20 min. Hakenberg et al. designed a phase-guided passive microfluidic batch mixing chip for RPA. The device can be fabricated through an inexpensive approach that integrates dry film resist technology and direct wafer bonding [114]. This detection assay relies on phase-guided fluid handling, resulting in direct fluorescence detection from the chip after a one-minute mixing sequence. RPA has been also used in a droplet microfluidic chip. The microfluidic digital RPA slip-chip has been employed for the simultaneous running of over 1000 nL-scale RPA reactions in parallel [115]. The amplification process starts by adding a chemical initiator to each reaction compartment. During the experiment, precise temperature control is not required owing to RPA tolerance of fluctuations in the incubation temperature ranging from 37 to 42 °C. The generation of an amplified target material is monitored by fluorescence. The performance of this platform was validated by the successful amplification of methicillin-resistant *S. aureus* genomic DNA. RPA has been growing in popularity in digital microfluidics. Recently, Kalsi et al. [116] performed RPA on a digital microfluidic device using magnetic beads and a pre-concentration unit. The sample with DNA is immobilised on the magnetic beads and then is introduced to a pre-concentration unit that interfaces with the digital microfluidic device. Next, the DNA-loaded beads are pulled through an immiscible oil/aqueous interface directly onto the digital microfluidic platform. The required temperature for amplification is just 39 °C. The final sample volume is 2 μL in a single step and the assay time is less than 30 min with a LOD of 10^4^ bacteria colony forming units (CFU)·mL^−1^.

### 5.2. HDA-Based Microfluidic Devices

HDA is an isothermal amplification reaction working optimally at 65 °C and relying on DNA helicase activity. DNA helicase separates complementary strands of double-strand DNA to allow hybridization of target specific primers. The primers are then extended using DNA polymerase to produce target DNA copies. Each of the resulting products can then be copied, leading to exponential amplification of the target. The simplicity and high sensitivity of the reaction means HAD has potential for use in a microfluidic platform [38]. Ramalingam et al. [100] developed a real-time HDA microfluidic chip using PDMS and glass. During the fabrication of this sandwich structure, the HDA primers are dried onto the glass surface of each microchamber. This method allows for simple multiplex analysis of one single sample. The microfluidic chip was verified by successful HDA of target DNA at 62 °C. HDA has been also applied on disposable plastic cartridge [117]. Despite the distinct advantages, there are still some drawbacks that limit its wide application. For example, HAD has been reported to be prone to sample contamination. The non-specific amplification products is a concern when using this method [118].

### 5.3. LAMP-Based Microfluidic Devices

Among the aforementioned methods, LAMP [14,119] was demonstrated to be quicker and more stable, more sensitive, and more specific for NA identification. LAMP-based approaches produce approx. 50-fold more amplicon than PCR-based methods [120]. Using LAMP, the amplification of medium- to long-range template strands of NA (130 < bp < 300) is possible [121].

Most importantly, LAMP can amplify NA in complex substrates even in the presence of contaminants that typically hinder PCR reactions, such as blood components [122] or food ingredients [123]. LAMP-based approaches have high specificity due to implementation of four to six different primers that bind to specific sites on the template strand [124]. LAMP amplification is conducted at temperatures between 60 °C and 66 °C employing the Bst polymerase enzyme and high strand displacement activity [121]. However, RNA targets need extra reverse transcriptase enzyme to transcript RNA into cDNA before the amplification step. A number of works have been conducted on monitoring LAMP amplicons using microfluidics. These detection methods for LAMP amplicons can be divided into end-point colorimetric detection and real-time detection.

#### 5.3.1. End-Point Colorimetric Detection

The colorimetric detection relies on production of magnesium pyrophosphate (Mg_2_P_2_O_7_) as a by-product of the reaction between deoxynucleotide triphosphate (dNTP) and magnesium sulfate (MgSO_4_). Magnesium pyrophosphate appears as a white precipitate in the reaction mixture, which increases its turbidity [13]. The presence of this component allows easy distinction of whether NA is amplified by LAMP. Although no extra instruments are needed, this approach relies on human interpretation of the colour. Different colorimetric dyes have been applied to detect the existence of amplicons, but these dyes should not hinder the amplification, and the colour change should be easily detected by the naked eye. The dyes that have been applied for bacterial LAMP amplicon detection are listed in Table 3.

Calcein and hydroxynaphthol blue (HNB) are the two frequently used dyes that do not interfere with the LAMP reaction [125]. Thus, these dyes can be added to the samples before starting the amplification reaction. Conversely, other dyes, such as propodium iodine, SYBR GREEN I, and Picogreen, have to be applied after amplification due to their inhibition effect on LAMP.

**Figure 4:**
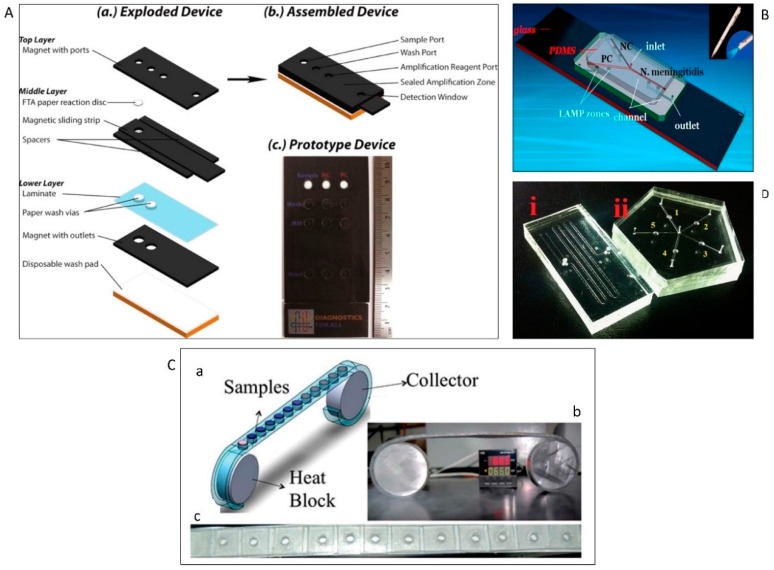
(**A**) Schematic illustration of sliding-strip device: (a) the exploded view and (b) the assembled device. (c) An image of a prototype concept device [130]. (**B**) Schematic depiction of the chip layout [129]. (**C**) Photograph of the cassette microfluidic device [131]. (**D**) Image of the airborne bacterial capture and LAMP system: (i) bacteria capture chip and (ii) LAMP chip [31]. Reproduced from mentioned reference with permission from the related journals.

Over the last few years, many papers reported the integration of different functions in a simple platform such as paper [132,133,134]. Connelly et al. designed a paper-based microfluidic device for identification of the *E. coli malB* gene [130]. Three layers of magnetic slips and a cellulose fibre network were used to construct this sliding-strip device, which enables the serial operation of cell lysis, DNA extraction, purification, LAMP amplification, and detection. SYBR GREEN I was used to detect the amplicons with a LOD of 5 cells in 80 μL of sample (Figure 4A). In another study, a hybridised paper/plastic microfluidic chip wa manufactured for the detection of *Neisseria meningitidis* applying calcein that showed a LOD of 3 DNA copies in a 26-μL sample (Figure 4B) [129]. A microfluidic cassette comprising of two aluminum reels and a plastic ribbon with an array of chambers was developed to identify *E. coli* (30 CFU/mL) and *S. aureus* (200 CFU/mL) using HNB and calcein detectors, respectively (Figure 4C) [131].

Although SYBR GREEN I, calcein, and HNB still attracted considerable interest in recent years, leading to rapid development of LAMP on-chip [126,127], there are drawbacks that should be addressed. One main limitation is the difficulty of detecting weak LAMP fluorescence in a few microliters of sample with the naked eye. Channels and reaction chambers with heights of more than 800 μm are possible solutions for higher sensitivity [135,136,137]. Jiang et al. developed a microfluidic chip that has a staggered herringbone mixer (SHM) structure to perform rapid and efficient airborne bacteria capture and high-throughput LAMP analysis [31]. Using deeper channels and calcein, the amplicons could be observed by the naked eye (Figure 4D).

Xia et al. reported another solution that optimized the concentration of calcein for LAMP reactions on a rotate-and-react SlipChip to increase the sensitivity [128]. Under the optimized LAMP conditions, *Bacillus cereus*, *E. coli*, *S. enterica*, *V. fluvialis*, and *Vibrio parahaemolyticus* were identified with the naked eye. The team achieved a fluorescent signal-to-noise ratio of about five-fold and a LOD of 7.2 copies/µL genomic DNA.

All the aforementioned methods and the most recent publications [126,127] confined their approaches to the application of Mg_2_P_2_O_7_ for naked-eye observation of the amplification; using gold nanoparticles (AuNPs) could be an alternative method. AuNPs depict a characteristic localized surface plasmon resonance absorption band (LSPR) in the visible spectrum of light, which depends on the interparticle space. The aggregation leads to a red shift originating a red-to-purple colour change [138]. Garrido-Maestu et al. reported microfluidic LAMP amplification using functionalised AuNPs for naked-eye detection of *Salmonella* spp. in food samples. This method achieved a very low LOD of 10 CFU/25 g [29].

#### 5.3.2. Real-Time Optical Detection

The main concept behind optical LAMP detection is the production of Mg_2_P_2_O_7_ as a side product of the LAMP polymerase reaction. Enhancement of magnesium pyrophosphate leads to the turbidity of the sample, which can be visualised with a turbidimeter [139], spectrophotometer [140], surface plasmon resonance (SPR) sensor [141], and real time [142] or fluorescent imaging by a charged coupled device (CCD) camera [143]. Amplicons can be enumerated by plotting turbidity against amplification time. Stedtfeld et al. designed a valveless microfluidic device to detect multiple genes, including *stx2* and *eaeA* of *E. coli* and *mecA* and *vicK* genes of *S. aureus* [144]. In this work, SYTO-81 dye was added to the reaction mixture before being loaded to the microchambers. The amplification was performed for an hour at a temperature of 63 °C. A LOD of 13 copies per sample (1 μL) was obtained using LED light at the bottom of each chamber.

Wang et al. reported the construction of a LAMP-based microfluidic device for the identification of methicillin-resistant *S. aureus* in applying a spectrophotometer [140]. Lysing, washing, and reaction chambers were integrated on a single chip. After thermal lysis of bacteria at 95 °C, the target DNA was recognized using specific probe-conjugated magnetic beads. Next, the target DNA was purified and LAMP reagents were added to the chamber. The amplicons were subsequently measured using a spectrophotometer. The entire sample treatment and amplification procedure was performed automatically, and a LOD of 10 fg/μL was achieved. CCD-based fluorescent imaging has also been applied for real-time monitoring of LAMP amplification to identify food- and water-borne pathogens (*Salmonella enterica*, *Cryptosporidium parvum*, *Campylobacter jejuni*, *Legionella pneumophila*, *Escherichia coli* O157:H7, *Vibrio cholera*). SYTO-81 was used as the florescent dye, and real time imaging was conducted for *C. jejuni* 0414 gene detection. A single copy of a gene was distinguished within only 19 min [143]. Chang at al. used an optical photomultiplier (PMT) for multiplex detection of *Streptococcus galactiae* and *Aeromonas hydrophila*. The target DNA was amplified and optically identified within 65 min with a LOD of 20 copies in a 25-μL sample [145]. Chiu et al. reported an SPR-LAMP-based chip. Single-layer graphene was deposited on the surface of the Au SPR chip to capture *Tuberculosis bacillus* DNA [146]. Although label-free optical detection modalities are attractive for developing real-time detection of LAMP amplicons, these approaches need bulky and expensive readers.

Zhou et al. [147] used CapitalBio RTisochip-A™ isothermal chip detection system for bacterial NA analysis in real time. This commercial platform was developed by CapitalBio Co. (Shanghai, China) and contains both LAMP amplification and an imaging system. The device can simultaneously detect 10 pathogenic bacteria in aquatic animals (*Nocardia seriolae*, *Pseudomonas putida*, *Streptococcus iniae*, *Vibrio alginolyticus*, *Vibrio anguillarum*, *Vibrio fluvialis*, *Vibrio harveyi*, *Vibrio parahaemolyticus*, *Vibrio rotiferianus*, and *Vibrio vulnificus*), with the LOD ranging from 0.40 to 6.42 pg per 1.414 μL and reaction time of less than 30 min. The CCD sensor has been the most common technique for real-time imaging [30,128,148]. Chen et al. reported a microfluidic in-gel loop-mediated isothermal amplification (gLAMP) for simultaneous detection of *E. coli*, *Proteus hauseri*, *Vibrio parahaemolyticus*, and *Salmonella* subsp [148]. The emitted fluorescence was evaluated with an inverted fluorescence microscope equipped with a CCD camera. This simple and easy-to-operate system achieved a LOD of 3 copies/µL, which is comparable to existing platforms and has potential for point-of-care applications.

## 6. Design Considerations for PCR Devices

### 6.1. Sealing

Microfluidic devices are typically sealed to contain the sample in a predetermined volume, avoiding uncontrolled spreading of liquids, preventing contamination and biohazards, and decreasing evaporation. Despite recent progress, sealing of these microdevices remains a complex and laborious process requiring specific equipment and protocols. In recent years, attention focused on providing robust, versatile, and reversible sealing solutions that are compatible with cell and molecular biology protocols. A wide range of techniques for sealing microfluidic chips have been outlined in past reviews [149,150,151]. Based on the materials that are used in chip fabrication and the limitations imposed by their application, sealing methods vary. PDMS and adhesive materials are the most commonly used materials in sealing devices for amplification.

PDMS, as the most popular material in the academic microfluidics community, is able to seal itself or other substrates both reversibly and irreversibly without an adhesive. Structured or flat layers of this elastomer can seal other flat materials such as silicon, glass, or plastics [152]. Uncured PDMS has been applied to seal inlets and outlets in PDMS-based microchips [136,153,154,155,156]. Although PDMS is an excellent material for rapid and easy sealing of many microfluidic devices, some drawbacks have restricted its wider usage. These limitations include the adsorption of hydrophobic samples, instability after surface treatment, swelling in organic solvents, water permeability, and inconsistency under high pressures [157,158].

**Figure 5:**
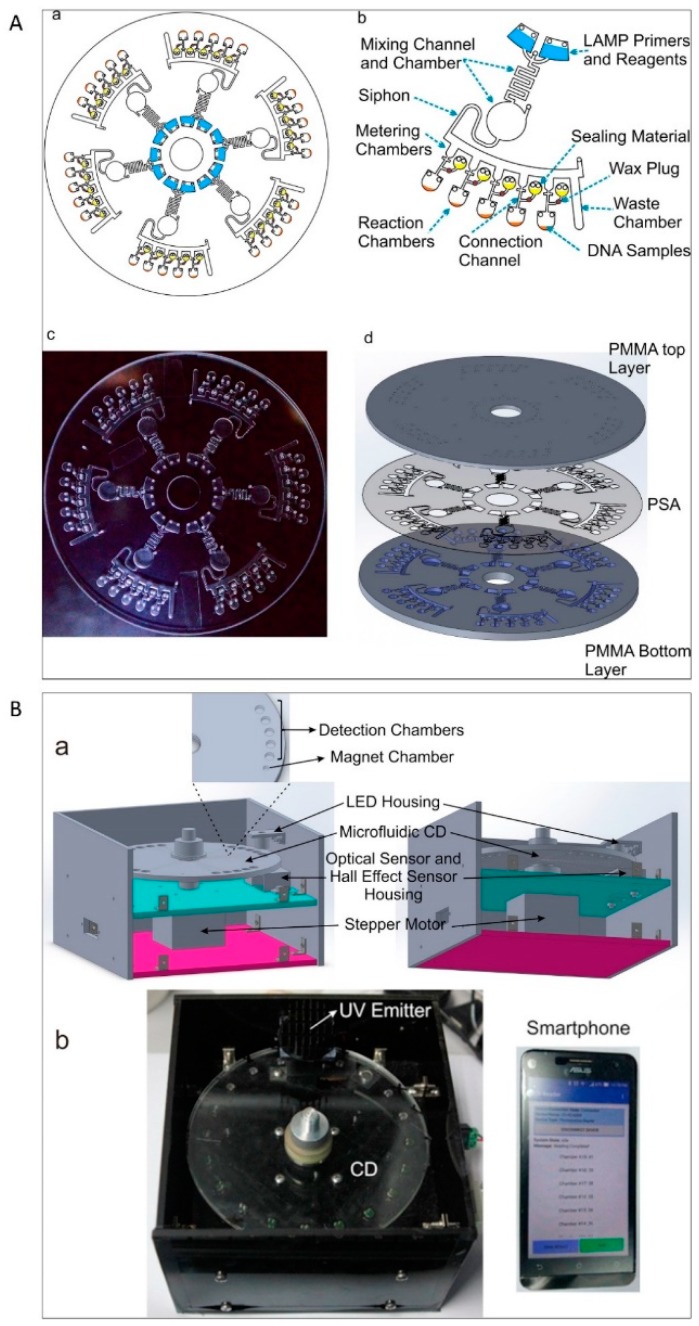
(**A**) (a) Schematic depiction of the centrifugal LAMP microdevice. (b) Top view of one unit of the microdevice. (c) Digital picture of the microdevice. (d) Schematic illustration of the microdevice consisting of PMMA and PSA. (**B**) (a) Three-dimensional (3D) model of the endpoint detection system. (b) Image of the endpoint detection platform with the application software running on a smartphone [65]. Reproduced from the mentioned reference with permission from the related journal.

An alternative approach is using adhesives for sealing microfluidic devices. Adhesive materials can overcome some of aforementioned problems. Pressure sensitive adhesive foil [83], polyolefin sealing foil [72], adhesive tape [80,86], adhesive sealing film [81], and UV adhesive [65] have been used. Sealing the inlets and ventilation holes with the above materials effectively prevented evaporation and contamination during the amplification reaction of bacterial NA. Sayad et al. investigated sealing solutions in an automatic centrifugal microfluidic platform for foodborne pathogen NA detection. In this system, the connection channel between the metering chamber and the amplification chamber was sealed to prevent liquid evaporation (Figure 5A,B) [65].

### 6.2. Valving

In addition to sealing, valving is the other critical issue that should be considered in designing a PCR device [159,160]. Under high temperatures, liquid sample may be lost due to thermocapillary pumping and evaporation. Thus, robust valving is required [70]. Generally, microfluidic valves can be categorized into two groups: passive and active. Passive valves are designed within the microdevice due to fluid flow or modified surface chemistry. Active valves can be classified based on working principles such as capillary valves [161], pneumatic siphon valves [162], and film valves [163]. However, most of these methods can incidentally leak vapor or fluid into an unwanted area due to the absence of a physical barrier. Active valves eliminate this shortcoming by employing a mechanical or external energy source to open and close the microvalve. A variety of techniques have been used for the active valve platforms, including magnetic [164], thermo-pneumatic [165], frozen liquid [70], hydrogel [166], and paraffin wax [167]. Some of these techniques have shown high potential for use mostly due to their low cost, simple operation, and biocompatibility. For example, Koh et al. employed an in-situ gel photopolymerization to form local gel plugs in an integrated plastic microfluidic device to detect *E. coli* O157 and *S. typhimurium* [97]. Using this technique, convective flow of the PCR mixture into other regions was remarkably minimized. In another study, Liu et al. used paraffin as single-use valving material in a disposable microfluidic device to identify *E. coli* [168]. This valve can hold a pressure of 40 psi in a “closed” position.

In another approach, Liu et al. designed thermally actuated valves using a PDMS-expandable microsphere composite [169]. Before the amplification, the valves were heated, expanded, and the amplification reactor was sealed. The valves were able to tolerate a pressure up to 200 kPa without any significant leakage. This device was successfully used for the detection of *E. coli*.

More recently, Brennan employed an elastomeric pinch valve in a microfluidic cartridge system for *E. coli* identification with a leak pressure of 340 kPa [170]. In another study, Huang et al. reported a simple valveless and air-insulated microfluidic chip for detecting a group of pneumonia-related pathogens such as *E. coli*, *Pseudomonas aeruginosa*, *Streptococcus pneumoniae*, *Klebsiella pneumoniae*, *Acinetobacter baumannii*, *Stenotrophomonas maltophilia*, *Haemophilus influenzae*, *Legionella pneumophila*, *Chamydiae pneumonia*, and *Mycobacterium tuberculosis* [73].

### 6.3. Detection

The common detection methods for amplicons are electrophoresis, real-time fluorescence analysis, turbidity, or colorimetry [13,125,131,138,171]. Among these methods, real-time fluorescence and colorimetric detection are the most widely used approaches for microfluidics due to their simplicity. Whereas fluorescent detection needs external equipment, colorimetric detection using metal indicators does not require extra detection instruments. Adding a metal indicator to the reaction solution can change the colour of the solution when the target gene is amplified. The colour change can be easily monitored with the naked eye. Oh et al. designed a centrifugal microfluidic device for multiplex identification of *E. coli* O157:H7, *S. typhimurium* and *V. parahaemolyticus* by loop-mediated isothermal amplification and colorimetric detection using Eriochrome Black T (EBT) [80]. EBT is a metal indicator that causes colour change by changing the Mg^2+^ concentration. Due to the reduction of the Mg^2+^ concentration over the amplification procedure, colorimetric detection can be a favourable choice for tracking the reaction. Similarly, this reagent was used for high-throughput screening of a group of foodborne pathogenic bacteria in another centrifugal device [81]. More recently, Sayad et al. reported a centrifugal microfluidic system with endpoint detection and identification of three pathogenic bacteria (*E. coli*, *Salmonella* spp, and *V. cholerae*), with eight strains each. Calcein, a synthetic fluorescein that emits a bright fluorescence, was used and further analysed via electronics interfaced with Bluetooth wireless transmission of the data to a smartphone [65].

## 7. Droplet-Based Microfluidics

Droplet-based microfluidic PCR is a technology that potentially provides fully programmable and automated PCR assays. Droplet-based microfluidics for the detection and the identification of pathogens presents several advantages over classical methods such as ultra-small sample volume, large number of droplet reactors, and ability to incorporate complex liquid handling protocols for these droplets. The large number of droplets allows the encapsulation of NA to follow a stochastic process. Single NAs are isolated from the bulk sample and are confined each in their own liquid compartment. A massively large number of individual droplets, even in the millions, could be evaluated, resulting in an extremely high throughput. Operations on droplets can be performed repetitively, allowing for more complex experimental protocols. Multiple manipulation tasks can be conducted on the same droplets [172,173,174] pre-and post-PCR: controlled droplet formation, merging, mixing with PCR reagents, splitting, sorting, and incubation [175,176]. Droplets are formed from two immiscible phases: the continuous phase (typically organic liquid-like oil in which droplets flow) and dispersed phase (the aqueous sample droplets). Aqueous droplets are commonly generated in a microchannel with the T-junction [177] or the flow-focusing configuration [178]. To date, the two droplet-based microfluidic technologies are continuous-flow and digital microfluidics. Compared to continuous-flow microfluidics, digital microfluidics implements the reaction protocols in a single droplet. Electrowetting and dielectrophoresis are common actuation techniques for digital microfluidics [33].

### 7.1. Continuous-Flow Microfluidic

Droplets are often in motion in continuous-flow droplet microfluidics. The physics of droplet formation and handling has been well studied [179,180,181]. Droplets can be incubated [182], split [183], and merged [184]. From the microbiological point of view, continuous-flow droplet-based microfluidics allows for the distribution of the large volume of aqueous suspensions of microorganisms in the order of millilitres or more into droplets with volume ranging from pico- to microlitres. The droplets can subsequently be manipulated automatically. Droplets can be formed at a frequency up to more than ∼10,000 Hz with a dispersity of less than 2% [185]. Droplet fluorescence can be analysed at a speed up to 250,000 droplets per second [186]. Instead of primer solution, some continuous flow droplet-based PCR systems used primer-modified beads [187] or agarose droplets [188].

To generate droplets, capillary tubes have often been employed. For instance, Dorfman et al. [189] used a PFA capillary, coiled around a cylinder heater to encapsulate PCR mixture in 1 mL droplets. Hartung et al. reported the droplet generation within Teflon FEP tube and T-connectors [190]. Markey also used PTFE tubing coiled around the aluminum cylinder heaters and T-junction to produce droplets [191]. In a different layout, Ohashi et al. applied the external magnet to move droplets containing hydrophilic magnetic beads through different temperature zones in a reaction chamber [192].

### 7.2. Digital Microfluidics

Digital microfluidics relies on stationary or semi-stationary droplets. This technology enables generation, manipulation, and monitoring of droplets carrying single or a bulk of NA in a highly parallel and high throughput process. A large number of droplets can be produced via the surface-assisted approach [193,194]. Hydrophilic wells or through-holes are patterned on the substrate and trap the sample solution into a stationary droplet array. Fluorinated oil is generally applied to prevent evaporation during the thermal cycling process. Beneyton et al. [195] used a high-throughput droplet-based microfluidic platform for detecting and sorting of *E. coli* based on the enzymatic activity of CotA laccase. The analysis/screening format of the system enabled the analysis of the enzymatic activity in droplets at a frequency of 1,000 Hz and active sorting of droplets at 400 Hz. After cell growth and protein expression inside the droplets, a fluorogenic reagent was inserted. Fluorescence-marked droplet was then sorted, recultivated, and identified based on colorimetric assays.

Although selective cultivation is useful to enrich the target species of bacteria prior to sequence analysis, this method is not successful for bacteria that are not cultivable. Lim et al. [196] used a culture-independent strategy for sorting microbial cells based on genomic content. The fluorescence signal generated in droplets during PCR was applied for sorting and analysis of the specific gene sequences. In another study, encapsulation in droplets allows for massive amplification of *E. coli* while maintaining sequence accuracy and uniformity [197].

In addition to high-throughput digital microfluidics, devices with the ability to facilitate and accelerate the amplification procedure have also attracted considerable attention. Easley et al. [198] used a microfluidic genetic analysis system with sample-in–answer-out capability for *B. anthracis* identification in blood samples. A single syringe pump delivered the sample and reagents into the glass-chip for NA purification. Elastomeric membrane valves were employed for the isolation of each functional region of the device. Purified DNA and PCR reagents then entered to the 550-nL chamber for PCR amplification.

Hua et al. [199] presented a digital microfluidic platform for quick multiplexed real-time PCR of methicillin-resistant *S. aureus* and *Mycoplasma pneumoniae*. Fast PCR thermocycling was achieved by periodically shuttling the sample droplet between two fixed temperature zones in an oil-filled cartridge. The cartridge was composed of a printed circuit board.

One of the possible methods to accelerate the amplification experiments dealing with samples with large concentration differences is providing a wide dynamic range using digital microfluidics [200,201]. A wide assay dynamic range would increase the speed of measurements by preventing sample serial dilution before PCR amplification. Most of the microfluidic digital PCR platforms use the strategy of increasing the number of compartmentalized microreactors to provide a wide dynamic range [194,202]. However, increasing the reaction number results in a significant challenge in the fabrication of high-density chambers. To overcome this hurdle, a multivolume digital PCR method has been reported, where multiple microreactors with different volumes are used. Compared with single-volume digital PCR platforms, the multivolume method considerably decreases the total number of reactors while preserving the same dynamic range [203]. To fabricate numerous chambers with a large volume range on one glass chip, complex multistep lithography and wet-etching techniques have been used to produce wells with different depths [204].

**Figure 6:**
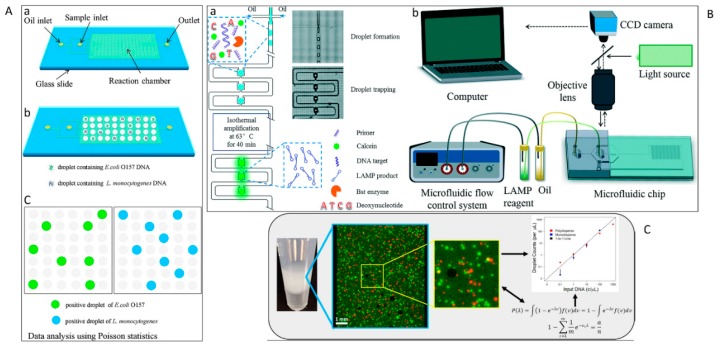
(**A**) Droplet digital PCR workflow: (a) fabrication of mineral oil saturated PDMS (OSP) microfluidic chip, (b) generation of droplets, (c) on-chip amplification followed by fluorescence readout, and (d) data analysis [96]. (**B**) (a) Schematic depiction of the assay for emulsion droplet array-based digital LAMP analysis. (b) eEperimental set up for result analysis [209]. (**C**) Multiplexing in ddPCR [210]. (Reproduced from mentioned reference with kind permission of related journal).

A critical concern in designing droplet digital platform is evaporation during thermal cycling. Bian et al. [96] used a mineral oil-saturated polydimethylsiloxane (OSP) chip for droplet digital PCR. The system provided droplet generation, amplification, and end-point fluorescence readout (Figure 6A) to identify *E. coli* O157:H7 and *L. monocytogenes*. Although the initial efforts led to sensitive detection of target sequences in bacteriology at single-molecule resolution, which is even higher than qPCR, there were still some drawbacks. For example, conventional digital microfluidics usually employs a relatively small number of fixed hard-wired electrodes. Consequently, the droplet manipulation operations were restricted to these electrodes patterned on the device. Over the last years, thin film transistor (TFT) electronics have been introduced as an ideal alternative [205]. These active matrix platforms have thousands of individually trackable electrodes allowing for simultaneous and independent manipulation of several droplets and running complex analytical processes. Kalsi et al. [206] presented a digital microfluidic system using TFT for detection of three genes that confer resistance in bacteria to antibiotics (*CTX-M-15*, *KPC*, and *NDM-1*). In this assay, automated dispensing protocols were applied to generate droplets with nL-volume-comprising sample DNA, reagents, and controls. The reagents were then mixed, and isothermal DNA amplification of droplets was performed. Positive amplification was measured by fluorescence.

Though digital platforms can generate large quantities of droplets automatically, there are still some practical drawbacks. For example, droplet-to-droplet coalescence may occur if they contact each other [207] during the heating process [208]. One of the possible solutions for this problem is the use of a droplet array. Ma et al. [209] presented a novel microfluidic device capable of arraying emulsion droplets and conducting digital LAMP of vancomycin-resistant *Enterococcus bacteria*. The system was a combination of an emulsion droplet formation device with a hydrodynamic trapping array. After preparation of target NA and LAMP reagents, they were digitized on-chip into water-in-oil droplets using a flow-focusing configuration. Subsequently, droplets were hydrodynamically sorted into a droplet array. The method was able to produce uniformly sized droplets with a variation of less than 3%. Successful LAMP amplification and fluorescence detection of positive droplets were recorded with a fluorescence microscope, Figure 6B.

Although digitization of amplification has led to improved time to detection and direct quantification of NA without a standard curve, these methods mostly are limited to laboratories with trained personnel and expensive equipment. To overcome these hurdles, Byrnes et al. [210] reported a polydisperse droplet emulsions approach with a statistical correction for *E. coli* detection. The assay provides accurate quantification of droplet digital PCR and reverse transcriptase droplet digital PCR which makes it more powerful compared to commercially available devices such as BioRad’s ddPCR. This method overcomes a few practical restrictions of the BioRad system. For example, since the measurement of droplets occurs regardless of size, there is no data loss due to improper droplet size. Also, it requires less equipment and time for running (Figure 6C).

Table 4 provides a brief list of the advantages and disadvantages of microfluidic-based amplification techniques which discussed above.

## 8. Microfluidic Sample Preparation for Amplification

To fully integrate an amplification protocol onto a microfluidic device with sample-to-answer capability, the following processing steps have to be integrated: cell lysis, NA extraction and purification, amplification, and amplicon detection [211,212]. Several devices were reported to complete cell lysis and DNA extraction with PCR using silica-based separations or magnetic beads for extraction [213,214]. However, due to the inhibition effect of silica and some magnetic beads on amplification, the DNA should be eluted, usually with ethanol, which is a strong PCR inhibitor as well.

Oblath et al. [215] used a monolithic aluminium oxide membrane (AOM) in an integrated microfluidic device. The device was successfully used for the identification of methicillin-susceptible *S. aureus* and methicillin-resistant *S. aureus* in saliva. AOM is a porous material that can be applied for the extraction of DNA. The amount of extracted DNA relies on the size of the AOM’s pores, pH, and salt concentration of the solution. In this approach, bacteria were first lysed by heating. The resulting solution was then injected to the chip and filtered via the AOM to extract the DNA. By adding PCR reagents to each well, the chip was ready to be thermocycled. Ethidium monoazide (EMA) is another commonly used material for differentiating live and dead bacteria. EMA is a DNA staining fluorescent dye that can enter into the broken cell walls of dead bacteria and intercalate into double-stranded DNA. Since the EMA cannot enter live cellular membranes, it can be used for labelling the DNA of dead cells within a population of viable and dead cells [216]. This marker has been used in various integrated amplification microfluidics to detect bacteria such as methicillin-resistant *S. aureus* (MRSA) [216], *E. coli*, *S. aureus*, *P. syringae*, *Enterococcus* sp., methicillin-resistant *S. aureus*, and coagulase-negative staphylococci [217], and live bacteria for periprosthetic joint infection [218].

Magnetic beads are the other group of material that can be used to eliminate the presence of inhibitors in the sample and to increase the collection efficiency of target DNA. Particularly, nucleotide probe-conjugated magnetic beads have been employed to capture specific DNA fragments [219]. Chao et al. [220] used magnetic beads in an integrated microfluidic device for *Helicobacter pylori* detection. In this experiment, the surfaces of the magnetic beads were modified with 16S rRNA probes to capture the conserved DNA region of *H. pylori*. After capturing the target DNA, SYBRs Green I, as a fluorescent dye, was added in the PCR step to intercalate into the DNA fragments, resulting in a measurable fluorescence intensity in the developed microfluidic system.

Another on-chip DNA purification technique is solid-phase based DNA collection. Ha et al. [221] employed an integrated thermoplastic microdevice for solid-phase based NA purification. The prepared chip consisted of three microchannels for washing solution, DNA purification, and amplification. Polycarbonate (PC) was the chip material. The surface of PC was treated with amine-bearing polyethyleneimine (PEI) to make it hydrophilic. After sealing the device, the microchannel walls were coated with epoxy-terminated poly(dimethylsiloxane) (PDMS) (epoxy-PDMS). Chambers functionalised with amine were used for capturing NA. The microdevice was successfully assessed by detection of genetically modified *E. coli* O157:H7. The same team also used glass beads to perform solid-phase based on-chip DNA purification to detect *E. coli* [222].

**Figure 7:**
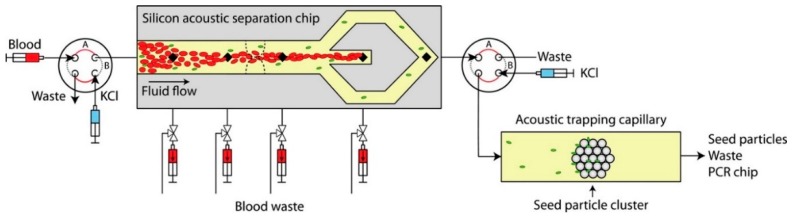
Bacteria were separated from red blood cells using acoustic separation, enriched, and then released to dry-reagent PCR chips for detection [224]. Reproduced from the mentioned reference with permission from the related journal.

Kim et al. [223] employed a solid phase reversible immobilization (SPRI) method for NA extraction in a high-throughput automated microfluidic system. This platform was used for whole-genome shotgun (WGS) sequencing of *M. tuberculosis* and soil micro-colonies. Another possible alternative to the aforementioned methods is the separation and enrichment of bacteria before amplification. Ohlsson et al. [224] developed an integrated microchip with acoustic separation, enrichment, and PCR detection of bacteria from blood. The blood sample was first processed in an acoustophoresis chip to remove red blood cells. Next, the remaining bacteria-containing plasma proceeded into a glass capillary where the bacteria were trapped and enriched onto suspended polystyrene particles. The trapped bacteria were subsequently washed and released into a polymeric device containing dried PCR reagents for amplification (Figure 7).

Recently, Ip et al. [225] used modified magnetic beads in an integrated microfluidic platform to identify live *M. tuberculosis* (TB). The device comprises four identical diagnostic sets including reaction chambers, positive chambers, negative chambers, and components for automatic liquid handling. Bacteria were trapped using magnetic beads modified with TB-specific markers. Since the magnetic beads capture both live and dead TB, propidium monoazide (PMA) was used in the second step for differentiating these cells. PMA is able to preferentially bind to dsDNA. This photoreactive dye is unable to penetrate the membrane of viable bacteria, so it can be applied for selectively capturing dead bacteria. After washing unbound bacteria and unnecessary reagents, bacteria were lysed and the resulting DNA was used for amplification and fluorescence detection. Yu et al. [226] used mannose-binding lectin (MBL)-coated magnetic beads in an integrated microfluidic system to isolate methicillin-resistant *S. aureus* and *E. coli*. MBL is a liver-derived serum protein with the ability to identify carbohydrate patterns on a broad range of pathogens, and particularly bacteria. Using this device, the entire process including bacteria isolation, on-chip amplification, and fluorescent signal detection is completed within 1 h.

## 9. Conclusions and Perspectives

Remarkable progress has been achieved in the field of microfluidics. Since its beginning, microfluidics applications are becoming increasingly more relevant to life sciences and medicine. One of the reasons for this achievement is due to the unique chemical and physical processes that occur on the microscale.

Microfabrication has much to offer to microbiologists, particularly in NA detection and identification without a major investment of time and resources. Since the first successful PCR in microfluidic devices was reported, a variety of techniques have been developed. The techniques provide rapid analysis, automation, high throughput, high specificity, high accuracy, portable type, low cost, and convenience. However, a number of time and space domain PCR devices are still unable to provide simple user interfaces. Fabrication facilities and skilled hands are required to handle these types of devices. The complexity is a significant barrier for PCR users, preventing them from adapting microfluidic devices. Only a few instances, such as the Lab Disk, leverage the advantages of microfluidics and provide the simple use. Future studies, particularly in spatial and transient PCR, should focus on making the microfluidic approaches easily accessible for users by simplifying of the fabrication process and consequently reducing the final price.

LAMP is a promising alternative to PCR and can reduce technical complexity for microfluidic devices. LAMP approaches have a higher robustness against temperature variations and inhibition compared to PCR. LAMP-based approaches still require vigilant optimization of loop primers for reproducible and sensitive target detection.

Microfluidics-based digital PCR has become a promising alternative to conventional amplification platforms, which allows absolute quantification with high precision without the requirement for standard curves. Digital PCR is based on a compartmentalization of NA molecules into individual volumes from bulk solution. Although digital PCR is being employed broadly, the impact of the technology could be extended. To attain this success, studies should be performed to allow for simple, automatic, high-throughput, and multiplexed digital PCR. Commercial integrated amplification platforms also have advantages, such as high throughput, simple operation, and low cross contamination. Even though experiment expenses could be decreased by a reduction in sample volumes, the cost advantages are not still significant enough for most end users. Consequently, simplification of digital PCR processes and reduction of required equipment is necessary, for example, by simplifying the sample pre-treatment method to shorten the assay time and further improve the detection limit.

Alternative approaches to NA detection, such as DNA arrays and next generation sequencing, have been used widely in the last years, providing another perspective for designing microfluidic-based detection systems. However, both arrays and sequencing methods often require a minimum amount of NA and thus need a PCR sample preparation step. Hence, integrating PCR and NA arrays or sequencing methods on the same microfluidic device could be an interesting approach for future research.

## Figures and Tables

**Table 1:** Summary of previous review articles addressing the microfluidic-based bacterial nucleic acid (NA) amplification approaches in chronological order.

Main Focus of the Review	Year
Review of few studies reporting microfabrication of PCR in microbiology [37]	2007
A comprehensive review paper of miniaturized isothermal nucleic acid amplification [38]	2011
A review on the general use of microfluidics in bacterial pathogens monitoring with less focus on amplification methods [39]	2014
A review of works based on centrifugal microfluidic platforms for NA detection and amplification in microbial samples [28]	2015
A review of a few studies on microfluidic PCR for bacterial pathogens identification [40]	2015
A comprehensive and systematic review of loop-mediated isothermal amplification-based microfluidics for pathogenic nucleic acid detection [32]	2016
A review of microfluidics based microbial engineering with a brief explanation of bacterial genotyping [19]	2016
A review of general PCR microfluidic devices [20]	2016
A review describing applications of droplet microfluidics in microbiology [41]	2016
A comprehensive review focusing on detection of microorganisms using microfluidic-based analytical approaches [42]	2018
A review of a few studies on micro-scale bacterial NA amplification [43]	2018

**Table 2:** Summary of key studies using centrifugal microfluidics to identify bacteria.

Target Bacteria	Sensitivity	Time (cycles)	Detection Technology	Heating Technology	Ref.
*Staphylococcus aureus*	Below 10 copies of DNA per well	110 min (50 cycles)	FAM-labeled hydrolysis probes; real-time fluorescence detection	Air mediated in commercially available PCR thermocycler	[78]
*Staphylococcus aureus*	Less than 7 copies per sample	17 min (10 cycles) primary PCR; 52 min (50 cycles) main PCR	FAM-labeled hydrolysis probes; real-time fluorescence detection	Air mediated in commercially available PCR thermocycler	[79]
*Salmonella enterica*	Amplification of one gene from single cell	8.33–20.83 min (20–50 cycles)	FAM-labeled hydrolysis probes; post-PCR fluorescence detection	Contact	[69]
*Bacillus anthracis, Bacillus cereus*	Not mentioned	53 min (35 cycles)	Off-chip (analysis of PCR products by gel electrophoresis)	Contact; with thermoelectric modules	[70]
*Listeria monocytogenes*, *Salmonella typhimurium*, EHEC, *S. aureus*, *Citrobacter freundii*, and *Campylobacter jejuni*	0.1 pg DNA per well for *Salmonella* and *Listeria*	Around 2 h (50 cycles)	FAM-labeled hydrolysis probes; real-time fluorescence detection	Air mediated in commercially available PCR thermocycler	[71]
*Escherichia coli*	Not mentioned	Around 54 min (30 cycles)	Agarose gel pre-stained with ethidium bromide	Convection of hot air and ambient air in commercially available PCR thermocycler	[72]
*Haemophilus influenzae*	Fluorescence sensitivity down to 100 CE with tmRNA	70 min (Isothermal)	FAM-labeled beacon probes; fluorescence detection	Non-contact infrared (IR) heating	[66]
24 Pneumonia-related pathogens	As few as 10 copies	45 min (Isothermal)	Real-time fluorescence detection	Contact	[73]
*Mycobacterium tuberculosis*	10^2^ colony-forming unit per millilitre	15 min (Isothermal)	Real-time fluorescence detection	Contact (printed circuit board heater)	[67]
*E. coli* O157:H7, *S. typhimurium*, and *Vibrio parahaemolyticus*	380 copies	60 min (Isothermal)	Colorimetric detection using eriochrome black T; naked eye	Contact	[80]
*E. coli* O157:H7, *S. typhimurium*, and *V. parahaemolyticus*	500 copies	60 min (Isothermal)	Colorimetric detection using eriochrome black T; naked eye	Contact	[81]
*E. coli*, *Salmonella* spp, and *Vibrio cholerae*	3 × 10^−5^ ng·μL^−1^	60 min (Isothermal)	Calcein colorimetric method; smart phone	Contact	[65]

**Table 3:** Different dyes employed for bacterial Loop-mediated isothermal amplification (LAMP) identification.

Dye	Colour Before Amplification	Colour After Amplification	Prevents LAMP	Ref
Hydroxynaphtol blue (HNB)	Violet	blue	no	[125]
Mixed-dye (HNB + SYBR Green I)	Orange-red	green	no	[126]
NeuRed	Light brown	pink	no	[127]
Gold nanoparticles	Purple	red	no	[29]
Calcin	Yellow	green	no	[31,128,129]
SYBR GREEN	Dark orange	green	no	[130]
HNB Calcein	Purple Yellow	Blue green	no	[131]

**Table 4:** Merits and demerits of microfluidic-based amplification platforms.

Platform	Complexity	Sample Volume	Assay Time	Throughput	Sensitivity	Utility
Serpentine [59]	-Several heaters and pumping are required-Complex channel design	0.35 μL	18 min	Low	0.031 pg/μL	On-site gene testing
Oscillating-flow [62]	-Pumping is required-Complex design-Low detection speed	2 μL	12 min	Low	10 DNA copies	On-the-spot analysis
Centrifugal [66]	-Elaborate designs and rotating platforms-Complex electronic components	40 μL	70 min	Low	100 CE with tmRNA	Clinical application
Lab disk [86]	-Very complex design-Long analysis time-Mostly unsuitable for multiplexing	4.8 μL of the sample plus preloaded primers and LAMP reagents	60 min	Low	2 × 10^2^ cells per μL	Nucleic acid diagnostics in resource-limited settings particularly in clinical stage
Array [102]	-Robotic liquid handling is required-Complex and expensive	20 uL	~60 min	High	High	Water distribution systems, clinical field
LAMP-based [139]	-Difficult naked eye detection in a few microliters of sample-Instruments for visualisation are required	600 nL	~70 min	Low	3 copies/μL	Applications in point-of-care settings
Droplet-based [201]	-Difficult droplet manipulation-Evaporation during thermal cycling-Droplet-to-droplet coalescence-Limited to laboratories with trained personnel-Expensive	Droplets ranging in diameter from ~1.5 to 13,117 μm with a median diameter of ∼56 μm (90 pL)	~70 min	High	0.682 copies/μL	Variety of settings for the quantification of nucleic acids in complex samples
